# Delayed Contrast-Enhanced MRI of the Coronary Artery Wall in Takayasu Arteritis

**DOI:** 10.1371/journal.pone.0050655

**Published:** 2012-12-07

**Authors:** Christopher Schneeweis, Bernhard Schnackenburg, Matthias Stuber, Alexander Berger, Udo Schneider, Jing Yu, Rolf Gebker, Robert G. Weiss, Eckart Fleck, Sebastian Kelle

**Affiliations:** 1 Department of Internal Medicine/Cardiology, Deutsches Herzzentrum Berlin, Berlin, Germany; 2 Philips Health Care, Hamburg, Germany; 3 Division of Magnetic Resonance Research, Department of Radiology, Johns Hopkins University, Baltimore, Maryland, United States of America; 4 Department of Electrical and Computer Engineering, Johns Hopkins University, Baltimore, Maryland, United States of America; 5 Department of Radiology, Centre Hospitalier Universitaire Vaudois, Center for Biomedical Imaging (CIBM) and University of Lausanne, Lausanne, Switzerland; 6 Department of Rheumatology, Charité Universitätsmedizin, Berlin, Germany; 7 Division of Cardiology, Department of Medicine, Johns Hopkins University, Baltimore, Maryland, United States of America; University of Virginia Health System, United States of America

## Abstract

**Background:**

Takayasu arteritis (TA) is a rare form of chronic inflammatory granulomatous arteritis of the aorta and its major branches. Late gadolinium enhancement (LGE) with magnetic resonance imaging (MRI) has demonstrated its value for the detection of vessel wall alterations in TA. The aim of this study was to assess LGE of the coronary artery wall in patients with TA compared to patients with stable CAD.

**Methods:**

We enrolled 9 patients (8 female, average age 46±13 years) with proven TA. In the CAD group 9 patients participated (8 male, average age 65±10 years). Studies were performed on a commercial 3T whole-body MR imaging system (Achieva; Philips, Best, The Netherlands) using a 3D inversion prepared navigator gated spoiled gradient-echo sequence, which was repeated 34–45 minutes after low-dose gadolinium administration.

**Results:**

No coronary vessel wall enhancement was observed prior to contrast in either group. Post contrast, coronary LGE on IR scans was detected in 28 of 50 segments (56%) seen on T2-Prep scans in TA and in 25 of 57 segments (44%) in CAD patients. LGE quantitative assessment of coronary artery vessel wall CNR post contrast revealed no significant differences between the two groups (CNR in TA: 6.0±2.4 and 7.3±2.5 in CAD; p = 0.474).

**Conclusion:**

Our findings suggest that LGE of the coronary artery wall seems to be common in patients with TA and similarly pronounced as in CAD patients. The observed coronary LGE seems to be rather unspecific, and differentiation between coronary vessel wall fibrosis and inflammation still remains unclear.

## Introduction

Takayasu arteritis (TA) is a rare chronic inflammatory disease of the large vessels and is characterized by granulomatous arteritis of the aorta and its major branches of unknown origin. Due to the chronic inflammatory process local artery stenosis can occur with its characteristic symptoms of claudication and blood pressure differences (“pulseless disease”) [1]. According to the American College of Rheumatology (ACR) classification criteria, diagnosis of TA is based on physical examination, laboratory findings and imaging studies [2].

Over the past decade, magnetic resonance imaging (MRI) has become established as a reliable non-invasive radiation free imaging technique for diagnosis of TA which seems to be comparable to the standard technique of x-ray angiography [3]. Typical signs of TA in MRI are vessel wall thickening and stenotic areas in large artery vessels. Moreover MRI provides the possibility to depict vessel wall edema as a sign of inflammation by T1 and T2 weighted images [4].

Coronary artery involvement may appear in 10 to 30% [5], [6] of patients with TA. Myocardial infarction can occur in about 10%, while angina pectoris as a typical symptom seems to be rare [7], but heart failure is common and develops in up to 60% [8], suggesting the importance of detecting coronary involvement in TA.

It is well known that inflammatory processes are key factors in the development and progression of coronary artery disease (CAD) [9], [10].

Recently, cardiac magnetic resonance (CMR) showed promising results in the detection of coronary artery stenosis [11]–[13] and describing coronary vessel wall alterations in patients with stable CAD post administration of gadolinium [14]–[16]. It is well known that coronary involvement occurs in inflammatory disorders of large arteries such as Kawasaki disease [17].

The aim of this study was to assess delayed contrast enhancement MRI of the coronary artery vessel wall in patients with TA and to compare these results to those in patients with known stable CAD.

## Methods

### Patients

We prospectively enrolled 9 patients (1 male, average age 46.2±13.4 years) fulfilling the American College of Rheumatology (ACR) criteria of TA. Diagnoses of all patients were made by specialists in rheumatology. Average history of TA was 13.8±1.5 years. Affected large vessels, recent and past immune suppressive therapy and further patient information are given (Table S1 and S2). Additionally we tested the following laboratory parameters on the day of the CMR scan: c-reactive protein (CRP), erythrocyte sedimentation rate (ESR) and blood count. In no patients was CAD known or documented in their history. In four patients with TA a coronary angiogram had been performed previously. Angiography was performed for suspected stenosis of the upper limb arteries or to evaluate aortic regurgitation.

In the CAD group 9 patients participated (8 male, average age 65±10 years); in all of them the diagnosis of significant CAD was based on x-ray angiography with stenosis ≥50%. There was no sign or suspicion of acute inflammation in any of the CAD patients. All CAD patients had clinical indications to undergo cardiac MRI.

Patients were not included if they were clinically unstable (e.g. unstable angina pectoris), had any medical history of anaphylactoid reaction or had any contraindication for MRI examination. The study was conducted in accordance with the ethical standards defined by local law. In general, written informed consent from patients was obtained before their inclusion in the study. In addition, all participants gave a signature, that their data could be used in anonymized form for scientific work.

### MR imaging protocol

The MR imaging protocol was followed as previously described [14]. In brief, all participants were examined in the supine position using a commercial 3.0 T whole-body MR imaging system (Achieva 3.0 T, Dual Transmit; Philips, Best, The Netherlands). A 32-element cardiac phased-array coil was used for signal reception. Cardiac synchronization was performed using a vector electrocardiogram [18]. All subjects underwent a standardized MR examination consisting of the following steps:

For localization of the heart in the three standard planes (axial, coronal and sagittal), a single shot SSFP sequence (multistack, multislice survey scan, TR/TE/flip angle = 3.8 ms/1.8 ms/20°was used. This scan was also used for localization of the respiratory navigator. Next, an axial mid-ventricular cine-segmented balanced SSFP sequence with 50 cine frames was performed to visually determine the individual rest period of the coronaries in diastole. Timing of the coronary MRI acquisition within the cardiac cycle (trigger delay) was then adapted to each patient's individual coronary artery rest period. For gating and subsequent correction of diaphragmatic motion during free breathing, a navigator (gating window: 5 mm) was placed at the dome of the right hemi-diaphragm.

A bolus of 0.1 mmol/kg body weight Gd-DTPA (low/single-dose) (Magnevist®, Bayer-Schering, Berlin, Germany) at an injection rate of 2 ml/s, followed by a flush of 20 ml of saline solution at the same rate, was then administered intravenously. Magnetic resonance coronary angiography (MRCA) of the right and/or left coronary artery system after contrast was performed with a previously described navigator-gated free-breathing and fat-suppressed, cardiac-triggered T2-prepared segmented k-space gradient echo imaging sequence [15]. The acquired voxel size was 0.7×1×3 mm^3^, reconstructed to 0.7×0.7×1.5 mm^3^.

The subsequent LGE scans of the coronary vessel wall were performed before and 39±5 minutes after contrast administration with a navigator-gated free-breathing and cardiac-triggered T1-weighted inversion prepared and fat-suppressed 3D black-blood segmented k-space gradient echo imaging sequence. In a previous study by our group we observed that LGE of the coronary vessel wall was already detectable 30–45 minutes after administration of contrast agent. The best results were achieved between 30 and 60 minutes, which seems to be similar for detection of LGE in the aorta [15]. Imaging after 30 minutes is needed due to the necessary wash-out of contrast from the blood-pool. Scans of the right and left coronary artery system were performed in the same orientation as the previously acquired T2prep images, to ensure adequate co-registration. Parameters of the sequence were: TR/TE/flip angle = 4.7 ms/1.6 ms/20°. Spatial resolution of the sequence was *1×1×2 mm^3^ and reconstructed to 0.7×0.7×1 mm^3^*. The inversion time (TI) of the inversion recovery sequence used to null blood was individually adapted in every patient. Subsequently a noise-scan was performed to assess noise [19].

### MRI image analysis

All images were interpreted by the consensus of two experienced observers. Left ventricular enddiastolic volume (LVEDV) and left ventricular endsystolic volume (LVESV) were estimated using the area length method in the absence of regional wall motion abnormalities (WMA). In the case of wall motion abnormalities Simpson's method was used for evaluation of LVESV and LVEDV. Left ventricular ejection fraction (LVEF) was calculated by subtracting LVESV from LVEDV. Late gadolinium enhancement (LGE) of the myocardium was estimated visually.

For analysis of coronary arteries a 15-segment model according to the American Heart Association segmentation was used [20]. Coronary arteries with at least 1 visible coronary segment on T2prep scan were included in the analysis. Coronary artery segments with stents or coronary artery bypass grafts (CABG) were excluded because of potential artifacts.

**Figure 1:**
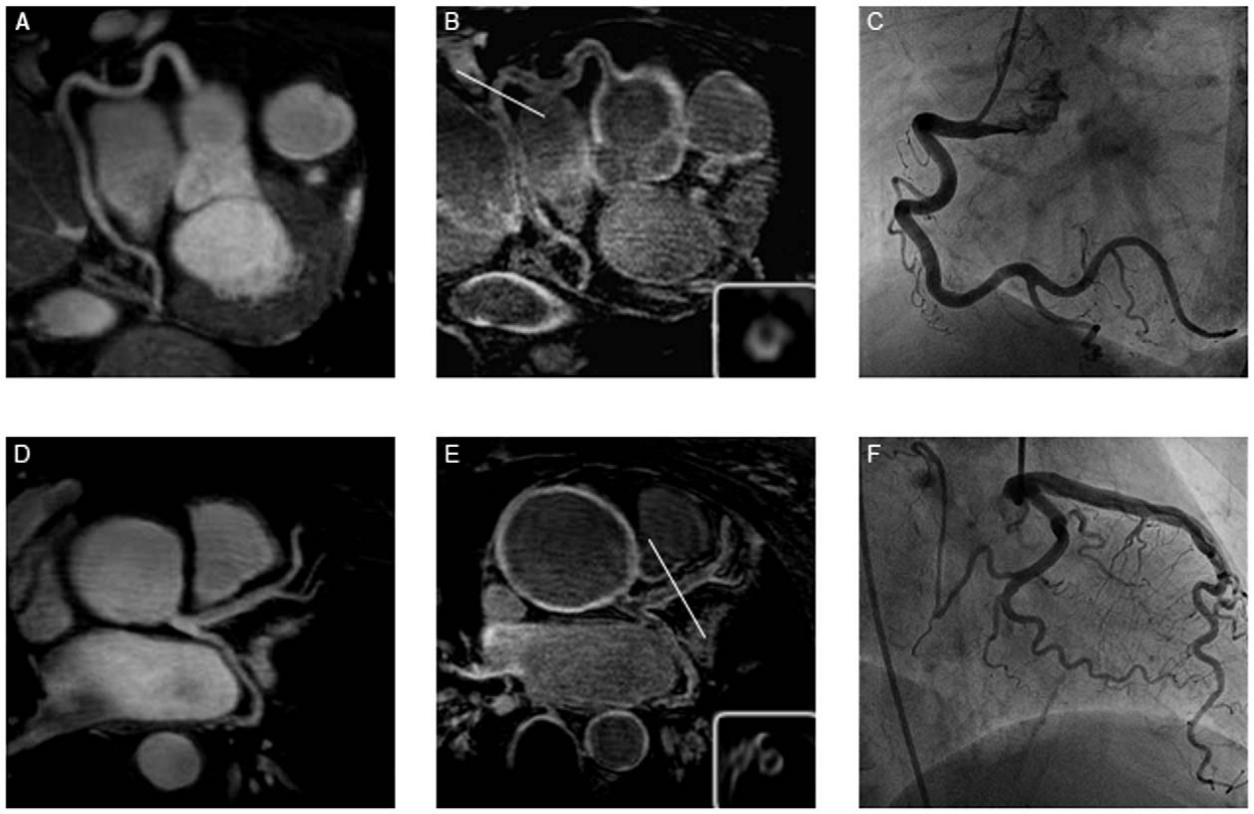
Representative T2prep images of the right coronary artery (RCA) (A) and the left main stem (D) in a patient with TA. B and E show the post IR scan of the same patient, revealing late gadolinium enhancement (LGE) of the coronary artery wall. C and F depict the corresponding x-ray angiography.

Firstly we assessed the coronary artery segments on a segment-by-segment basis as visible or nonvisible using the T2prep images by consensus of two experienced readers. All coronary arteries with LGE were assigned to the corresponding coronary arteries seen on T2prep images (Figure 1 A, D, G) and the number of enhanced segments was evaluated. For quantitative measurement we used View Forum software (Philips). The same procedure was performed for the visible parts of the ascending aorta. To determine the signal intensity (SI) of blood a contour was manually drawn in the ascending aorta. SI of coronary wall was assessed in each segment twice and the average was calculated. Noise was measured in the noise scans by drawing a contour manually. Signal to noise (SNR = (SI_wall_ or SI_blood_)/SD of noise) ratio was estimated with SNR = SI/SD of noise. Contrast enhancement within the coronary wall and the aortic wall was defined as the contrast to noise ratio (CNR). CNR = (SI_wall_-SI_blood_)/SD of noise [21].

In the TA group quantitative measurements of one patient had to be excluded due to inaccurate noise scans.

### Invasive coronary angiography

In four patients with TA (patient 1, 2, 4 and 5), x-ray coronary angiography was available. In all of these patients CAD was excluded.

In the CAD patients, clinically indicated invasive X-ray coronary angiography was performed within 6 months of MRI imaging using a standard Judkins technique.

### Statistics

Statistical analysis was performed using SPSS 18.0 for Windows (SPSS Inc.). All parameters were given as mean ± standard deviation. Analysis with paired student *t*-test was used. A non-parametric statistical test (Mann-Whitney U test) was used to compare presence of coronary LGE and measurements of SNR and CNR between the TA and CAD subjects. A non-parametric statistical test (Wilcoxon-signed rank test) was used to compare measurements of SNR and CNR within the TA and CAD subject groups. The results were assumed to be statistically significant with a p-value <0.05.

## Results

Two patients in the TA group had to be excluded from quantitative analysis, one due to discomfort during MRI scan and one because of inadequate scan quality. We also had to exclude the results of one patient in the CAD group owing to deficient noise scan.

The laboratory diagnostic tests in the TA group showed a slightly elevated CRP in 5 patients; overall CRP (mg/dl; normal range <0.5) was 2.3±2.8. The blood count was normal: in detail average white blood cells (WBC) count (K/µl, normal range <10) was 8.84±2.9 and slightly elevated only in three patients (Table S1).

### Left ventricular function and scar imaging

All patients in the TA group showed normal LVEF without WMA. No myocardial LGE was detected. Furthermore all patients had normal cardiac parameters (**Table 1**). The numbers of affected vessels separated for each TA patient are given in Table S2.

**Table 1:** Cardiac parameters of patients with Takayasu arteritis (TA) or coronary artery disease (CAD).

	TA (N = 9)	CAD (N = 9)
**age ± one SD (years)**	46.1±13.4	65±10
**male**	1	8
**left ventricular parameters**		
**LVEF (% ± SD)**	61.7±4.6	55.9±7.8
**LVEDD/mm ± SD)**	49.8±4.4	52.4±4.7
**LVESV (ml ± SD)**	52.4±10.4	75.6±31.9
**LVEDV (ml ± SD)**	136.3±22.7	165.7±40.4
**WMA at rest (# of pts.)**	0	4
**LGE (# of pts.)**	0	3
**coronary artery segments imaged**	28	25

CAD patients had a normal LVEF and normal cardiac parameters as well. In three patients myocardial LGE was present; two of them showed WMA. Overall, four patients had WMA at rest (**Table 1**).

### Late gadolinium enhancement (LGE) of the coronary artery vessel wall in TA and CAD patients

Twenty-eight of 50 visible segments (56%) in the TA group showed LGE (**Figure 1**). In these 28 coronary segments with LGE, quantitative assessment revealed a SNR of 18.4±6.7 (range: 10.5–26.8) and a CNR of 6.0±2.4 (range: 3.1–9.2).

**Figure 2:**
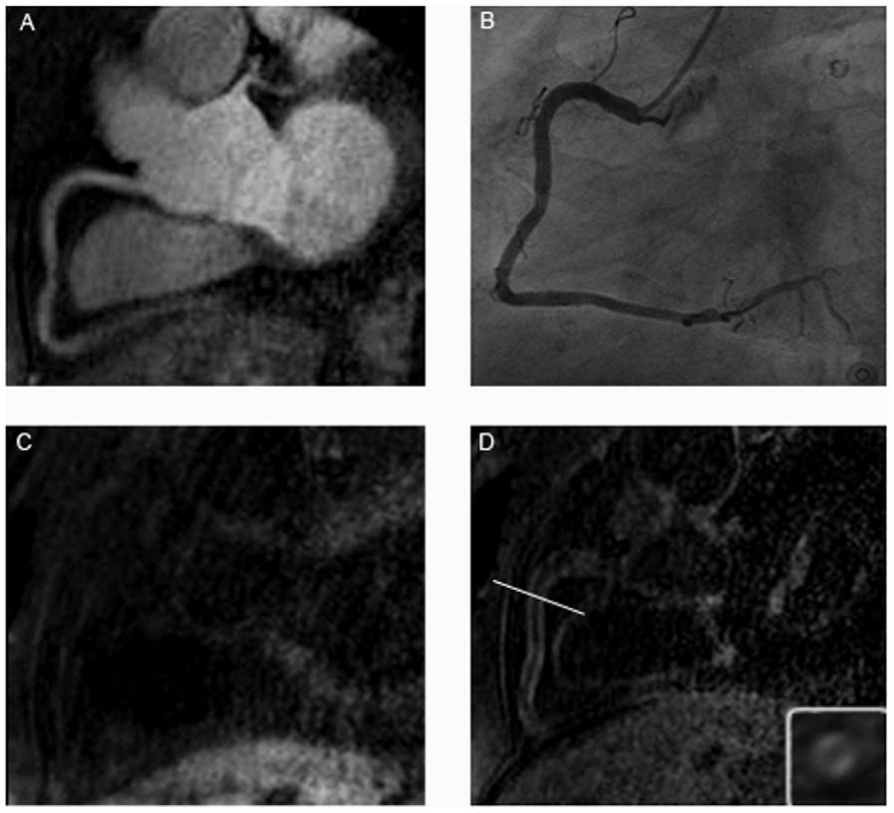
Figure A shows the T2prep image of the RCA in a patient with CAD, while B represents the x-ray angiography and C the IR scan pre and D the IR scan post gadolinium administration in the same patient. At the bottom right of Figure 1D is the enlarged cross sectional view of the appended IR scan.

**Figure 3:**
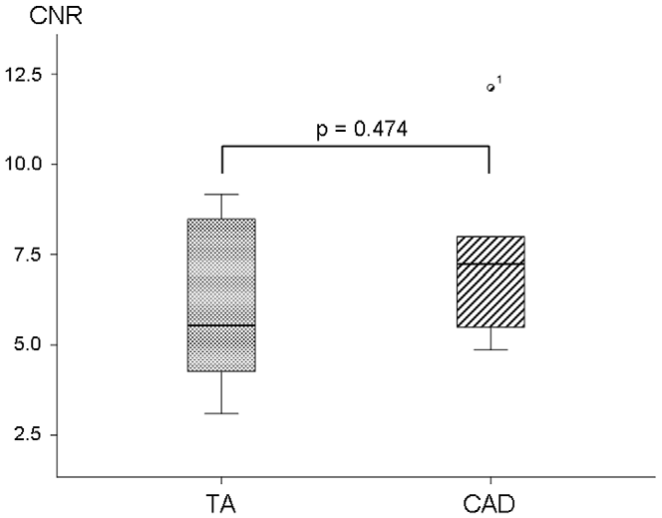
No significant difference of coronary CNR post contrast was seen between the patients with TA and those with CAD.

In the CAD group LGE was observed in 25 of the visible 57 segments (44%) (**Figure 2**). The quantitative analysis showed a SNR of 10.7±2.2 (range: 8.5–15.3) and a CNR of 7.3±2.5 (range: 4.9–12.1) (**Table 2**). Statistically there was no difference (p = 0.474) in LGE between the two groups (**Figure 3**).

**Table 2:** Quantitative MRI measures of signal-to-noise ratios (SNR) and contrast-to-noise-ratios (CNR) of patients with TA or CAD.

	TA (N = 9)	CAD (N = 9)	p-value
**LGE coronary artery wall**			
	6.0±2.4	7.3±2.5	0.474
	18.4±6.7	10.7±2.2	0.015
**LGE aortic wall prior Gd**			
	5.3±4.5	2.5±2.3	0.024
	8.8±6.5	7.7±3.7	0.092
**LGE aortic wall after Gd**			
	13.5±5.7	4.7±2.4	0.001
	26.5±9.6	7.7±3.7	0.001

**Figure 4:**
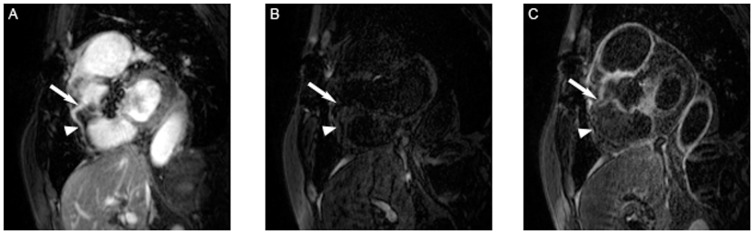
Figure A shows a T2prep image of the RCA in a patient with TA, while B represents the IR scan pre- and C the IR scan post- gadolinium administration in the same patient. The white arrow indicates the area of localized coronary artery vessel wall enhancement in C, the dotted arrow the coronary segment without LGE of the coronary vessel wall post contrast in the same coronary artery.

No increase in signal intensity of the coronary vessel wall was observed prior contrast administration, either in patients with TA (**Figure 4**) or in CAD patients (**Figure 2**).

### Late gadolinium enhancement (LGE) of the aorta in TA and CAD patients

**Figure 5:**
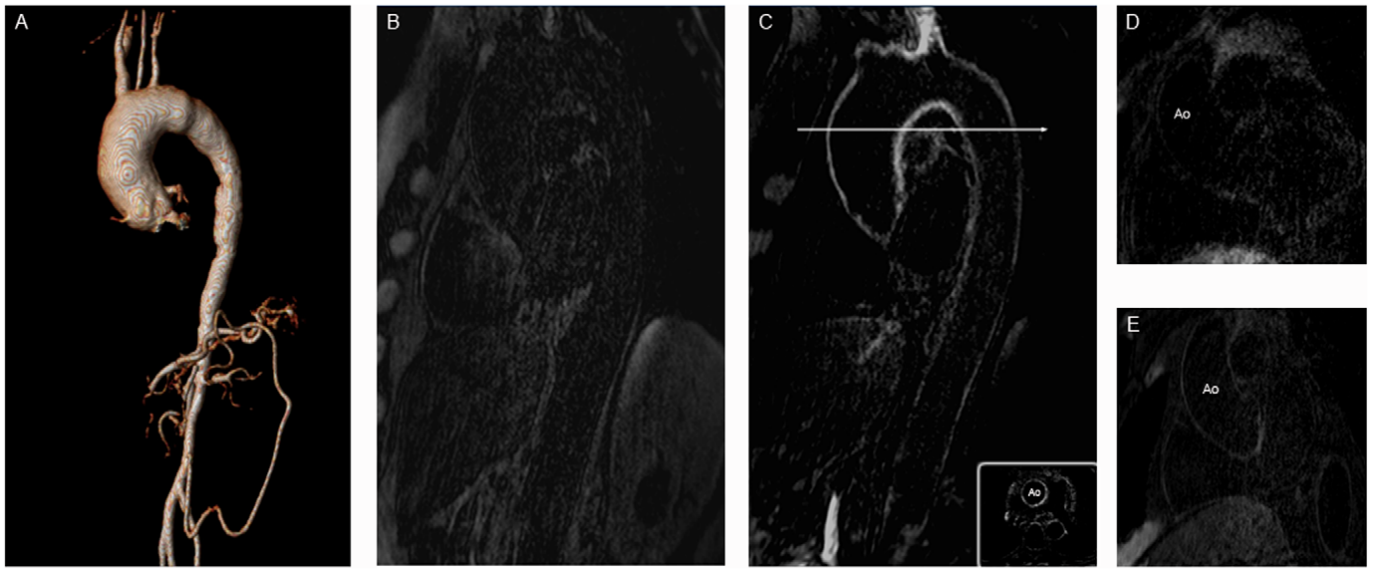
A shows the 3D reconstruction of a MR angiography and demonstrates a typical aneurysmal lesion of the ascending aorta caused by prolonged TA. Figure B shows an IR scan of the aorta in the sagittal plane before contrast administration in a TA patient. C depicts the same aorta post gadolinium application with reduced cross sectional view at the bottom. Exemplary IR image of the ascending aorta before contrast application in a patient with stable CAD (D). E represents the same aorta post contrast administration.

In the TA group quantitative assessment of the aortic wall demonstrated a significant increase of SNR and CNR post contrast administration (**Figure 5**). Before contrast agent we estimated a SNR of 8.8±6.5 (range: 0.0–19.0) and a CNR of 5.3±4.5 (range: 0.0–12.2); 39±5 minutes after gadolinium administration we observed a significant augmentation of SNR (26.5±9.6; range: 16.5–43.9) and CNR (13.5±5.7; range: 7.2–25.0). In the CAD group the estimation of SNR and CNR prior to contrast application was 4.1±3.1 (range: 0.0–7.1) and 2.5±2.3 (range: 0.0–5.2). After contrast application we assessed a SNR of 7.7±3.7 (range: 0.0–11.1) and a CNR of 4.7±2.4 (range: 0.0–6.7) (**Table 2**).

**Figure 6:**
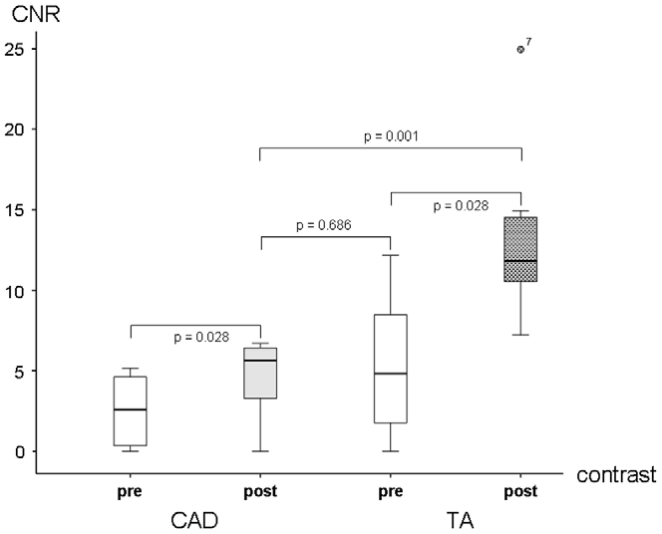
Both groups showed a significant increase of CNR of the aortic wall after contrast application with p = 0.028 in each group. The rise after contrast was significant higher in the TA than in the CAD group (p = 0.001). Interestingly there was no difference between post contrast in CAD compared to pre contrast in TA (p = 0.686) and no difference could be observed between the two pre contrast CNRs (p = 0.195).

Vessel wall enhancement of the aorta differed significantly before (p = 0.028) and after contrast application (p = 0.001) (**Figure 6**).

A statistically significant relationship between systemic inflammatory parameters and local coronary LGE or aortic LGE was not observed with this small number of patients.

## Discussion

In our study we demonstrated that late gadolinium enhancement (LGE) of the coronary wall occurs in patients with TA (without clincially overt coronary artery problems), which may indicate inflammation and/or fibrosis. We found no difference in quantitative measurements of coronary artery vessel wall LGE between patients with TA and patients with known CAD.

TA is a rare inflammatory disease with unknown etiology, mostly affecting women, and marked by panarteritis of the aorta and its major branches. In our population, 8 of nine patients with TA were women. Affected vessels become thickened, fibrotic and susceptible to thrombotic lesions. Due to these pathogenic processes the vessels may develop stenotic or aneurysmal areas.

Diagnosis of TA is based on clinical presentation, physical examination, laboratory findings and imaging modalities. Patients present with systemic non-specific clinical symptoms, such as fever, fatigue, weight loss and arthralgia.

So far there are no specific laboratory markers; common inflammatory markers such as CRP or ESR may be increased but showed no ability to distinguish between healthy patients and those with TA [22]. However, in a recent retrospective analysis of surgery versus endovascular intervention in TA patients it was shown, that biological inflammation at the time of revascularization increased the likelihood of complications [23].

Thus the diagnosis of TA still represents a challenge and imaging modalities have a rising impact and are crucial for the diagnosis. Increasing use of MRI for diagnosis of TA was observed during the last decade. The combination of T2 weighted images and contrast enhanced 3D magnetic resonance angiography (MRA) provides high-resolutional information on edema [4], [24], wall thickening and aneurysmatic formations and so represent an excellent alternative to conventional x-ray angiography. It was shown that after contrast agent application marked aortic arterial wall enhancement was observed in patients with TA [25] who underwent MRI.

Heart failure predicts the long-term prognosis of TA and seems to be the most common cause of death [26]–[28]. It is known that coronary involvement could occur in TA and three different pathological types of coronary artery involvement are known: occlusion or stenosis of the coronary ostium or the proximal segments; coronary arteriitis with diffuse or focal pattern and finally coronary aneurysm [29]. Most frequently the proximal segments of coronary arteries, seem to be affected, especially the left main stem (LM) and the left anterior descending artery (LAD) [29].

Besides TA, which presents as an inflammatory disease, it is widely accepted that inflammation plays a key role in the initiation and maintenance of atherosclerosis [10]. In recent studies it was possible to show that LGE is present in coronaries in CAD patients with stable disease [14]–[16] as well as in acute myocardial infarction [30], while no coronary LGE was observed in healthy subjects [14], [15]. However, gadolinium-based contrast agents are unspecific and the mechanism leading to contrast enhancement of the vessel wall and the potential differentiation between fibrosis and inflammation have not been fully investigated.

To our knowledge, this is the first study of assessment of coronary artery wall with MRI in patients with TA. Our findings suggest that late gadolinium enhancement (LGE) of the coronary wall occurs in patients with TA, which may indicate inflammation and/or fibrosis. Before contrast administration we did not find any coronary vessel wall enhancement, either in TA or in CAD patients.

Surprisingly there was no difference in quantitative measurements post contrast between patients with TA and patients with known CAD. The impact of these findings is still unclear; mostly coronary involvement in TA is not evident until the onset of typical clinical symptoms and is considered during autopsy [29]. No myocardial scar or WMA abnormalities at rest were found in patients with TA as evidence for prior myocardial infarction. Hence, we cannot predict whether coronary inflammation/fibrosis may be accountable for myocardial ischemia or cardiac failure in TA, although there are case reports about acute myocardial infarction in patients with the disease. Besides this, pathological reports have shown the incidence of myocardial inflammation [31]. Our study reported a subclinical involvement of the coronary vessel wall in TA patients. All TA patients were asymptomatic from a cardiac viewpoint, therefore, evidence of coronary vessel wall enhancement might hold a potential for a surrogate of subclinical cardiovascular disease, especially when cardiovascular risk assessment is not supported by presence of classical atherosclerotic risk factors. Dependent on further studies, evaluation of coronary vessel wall enhancement might serve as a potential therapeutic target to reduce coronary vessel wall inflammation in TA patients in the future.

We observed LGE in the aortic wall in all TA patients. Our findings are consistent with those of Desai et al. [21], who also reported increased CNR of the aortic wall after contrast application. All TA patients showed elevated aortic signal intensity before contrast application, and so CNR assessment was possible even before contrast application. The reason why we were able to perform quantitative measures of the aortic wall before contrast administration might be related to the larger size of the aortic wall (thicker walls) relative to the coronary artery vessel walls in which we were unable to measure CNR pre contrast. We found a significant increase of CNR after gadolinium administration, which may indicate inflammation or fibrosis of the aortic wall. The mechanisms which induce this LGE are still unknown, because histological confirmation is rare. Furthermore it is unclear whether LGE and CNR assessment may serve as indicators for response to therapy. All of our patients had a long duration of disease with long-term immunosuppressive therapy; therefore it was not possible to distinguish between enhancement in active and stable disease.

Recently an animal model showed the feasibility in specifically detecting atherosclerotic lesions and their progression or regression in MRI. Further studies are necessary, but these results are promising for future studies [32].

### Limitations

Our study is limited due to the small number of patients related to the rareness of TA. Therefore, a correlation between inflammatory laboratory markers and LGE of the coronary and aortic vessel wall was not possible.

On average, TA patients were around 20 years younger than CAD patients. In addition, most of the TA patients were women while most of the CAD patients were men. Therefore, a direct comparison of a gender- and age-matched group of patients with TA and CAD was not possible and not aim of this feasibility study, in which findings in patients with two different illnesses (TA and CAD) were compared. Future studies should evaluate the specificity of coronary LGE by testing if coronary vessel wall LGE can be detected in young women without a history or signs of an inflammatory vessel disease or stable CAD.

In addition it would be helpful to perform the same procedure in patients in acute and stable disease to discover whether differences exist and may be influenced by therapy.

### Conclusions

We showed that LGE of the coronary vessel wall can be detected by MRI and is common in patients with TA. In addition, the prevalence and amount of coronary enhancement after gadolinium (CNR) in TA seems to be similar to that in patients with CAD, while the impact of these findings in patients with different types of disease is unclear.

The observed coronary LGE in patients with TA or CAD seems to be rather unspecific, and differentiation between coronary vessel wall fibrosis and inflammation still remains unclear.

## Supporting Information

Table S1 **Shows the details of TA patients' characteristics and their medication history with immunosuppressive medication.**
(TIF)

Table S2 **Affected vessels separated for each TA patient.**
(TIF)
